# Supplementary material for: Single-cell transcriptomic analysis identifies the conversion of zebrafish Etv2-deficient vascular progenitors into skeletal muscle
Source: Nat Commun. 2020 Jun 3;11:2796. doi: 10.1038/s41467-020-16515-y (PMC7271194; doi:10.1038/s41467-020-16515-y)
Supplement: Supplementary file 2 — Supplementary Information [file 41467_2020_16515_MOESM2_ESM.pdf]

Supplementary Information

Single-cell transcriptomic analysis identifies the conversion of zebrafish Etv2-deficient vascular progenitors into skeletal muscle

Chestnut et al.

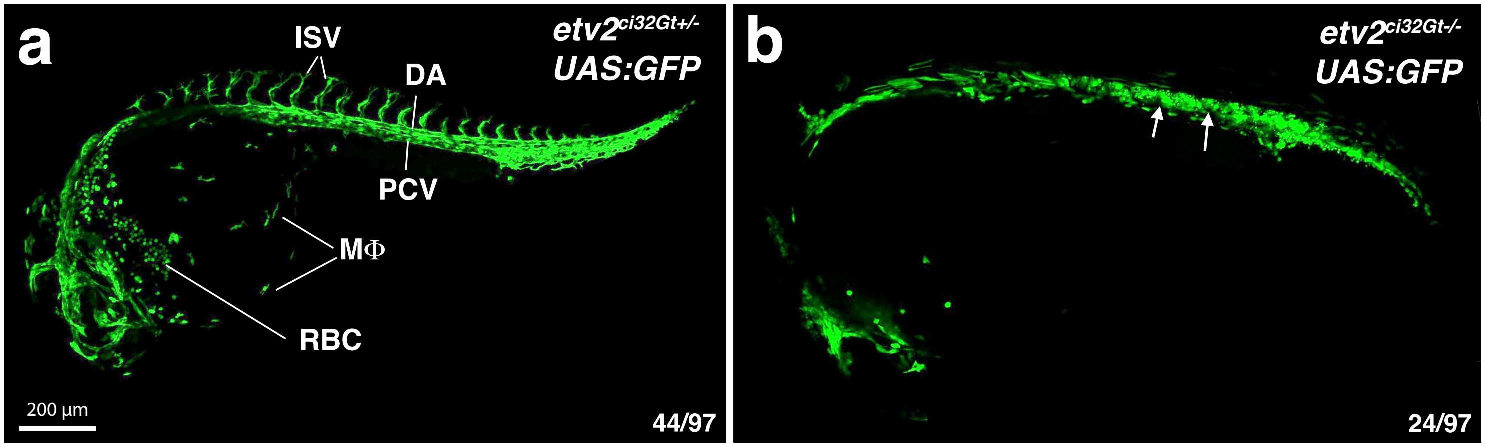

Supplementary Figure 1. Comparison of $\mathit{etv2}^{ci32Gt+/-}$ and $\mathit{etv2}^{ci32Gt-/-}$ embryos in *UAS:GFP* background at 25 hpf. (a) GFP expression is observed in all vascular endothelial cells, including the dorsal aorta (DA), posterior cardinal vein (PCV), intersegmental vessels (ISV), as well as red blood cells (RBC) and macrophages (MΦ). **(b)** Vascular progenitors fail to coalesce into vascular cords in $\mathit{etv2}^{ci32Gt-/-}$; *UAS:GFP* embryos (arrows). ISVs and macrophages are absent. Magnified view of the same embryos is shown in Fig. 3a,b. Numbers in the lower right corner show embryos which displayed the phenotype out of the total number of embryos obtained in an incross of $\mathit{etv2}^{ci32Gt+/-}$; *UAS:GFP* parents in two replicate experiments.

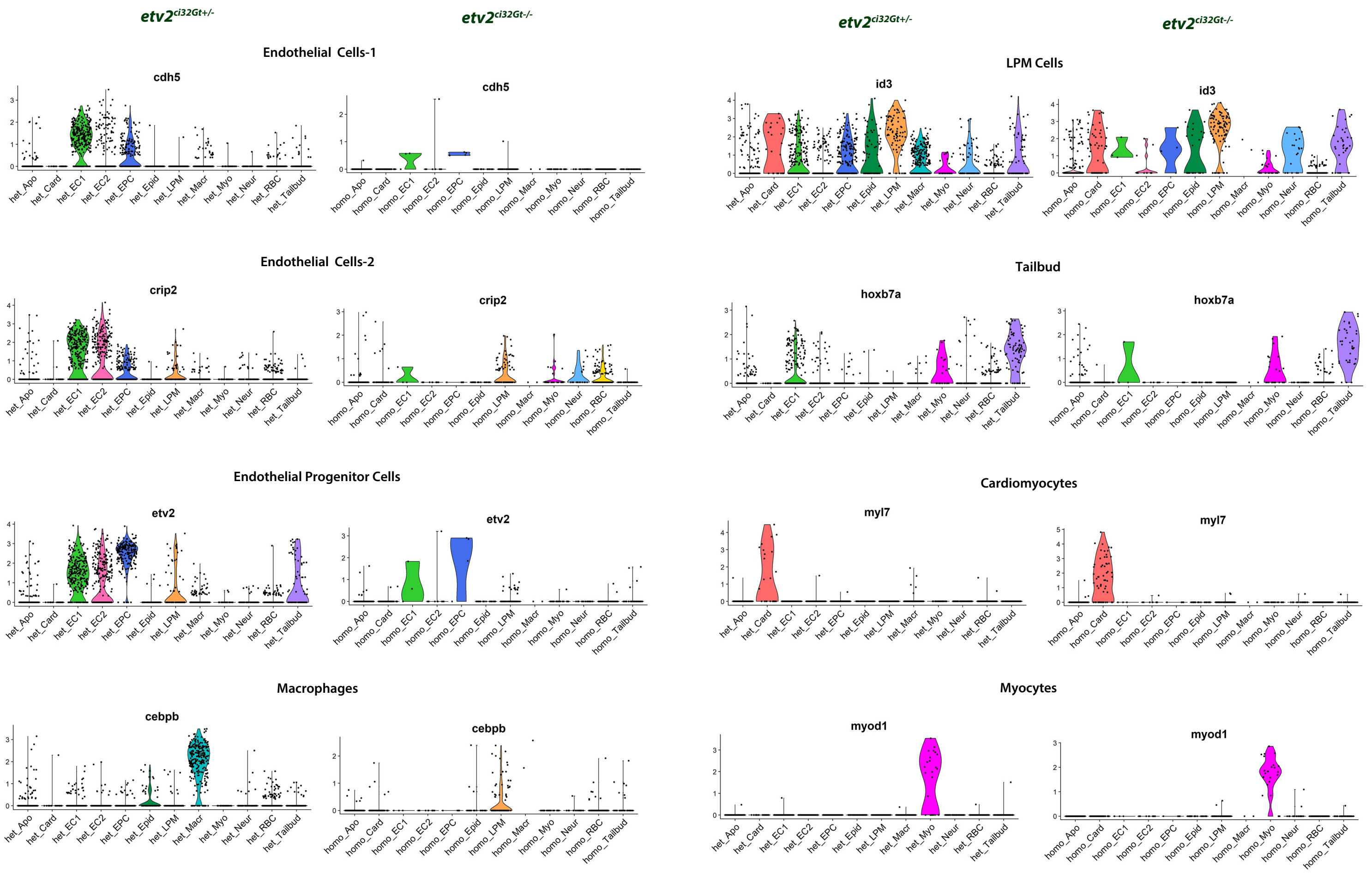

Supplementary Figure 2. Violin plots of top marker genes in different cell populations of *etv2*$^{ci32Gt+/-}$*; UAS:GFP* and *etv2*$^{ci32Gt-/-}$*; UAS:GFP* embryos. Selected markers and cell populations match the t-SNE graphs in Figure 1c.

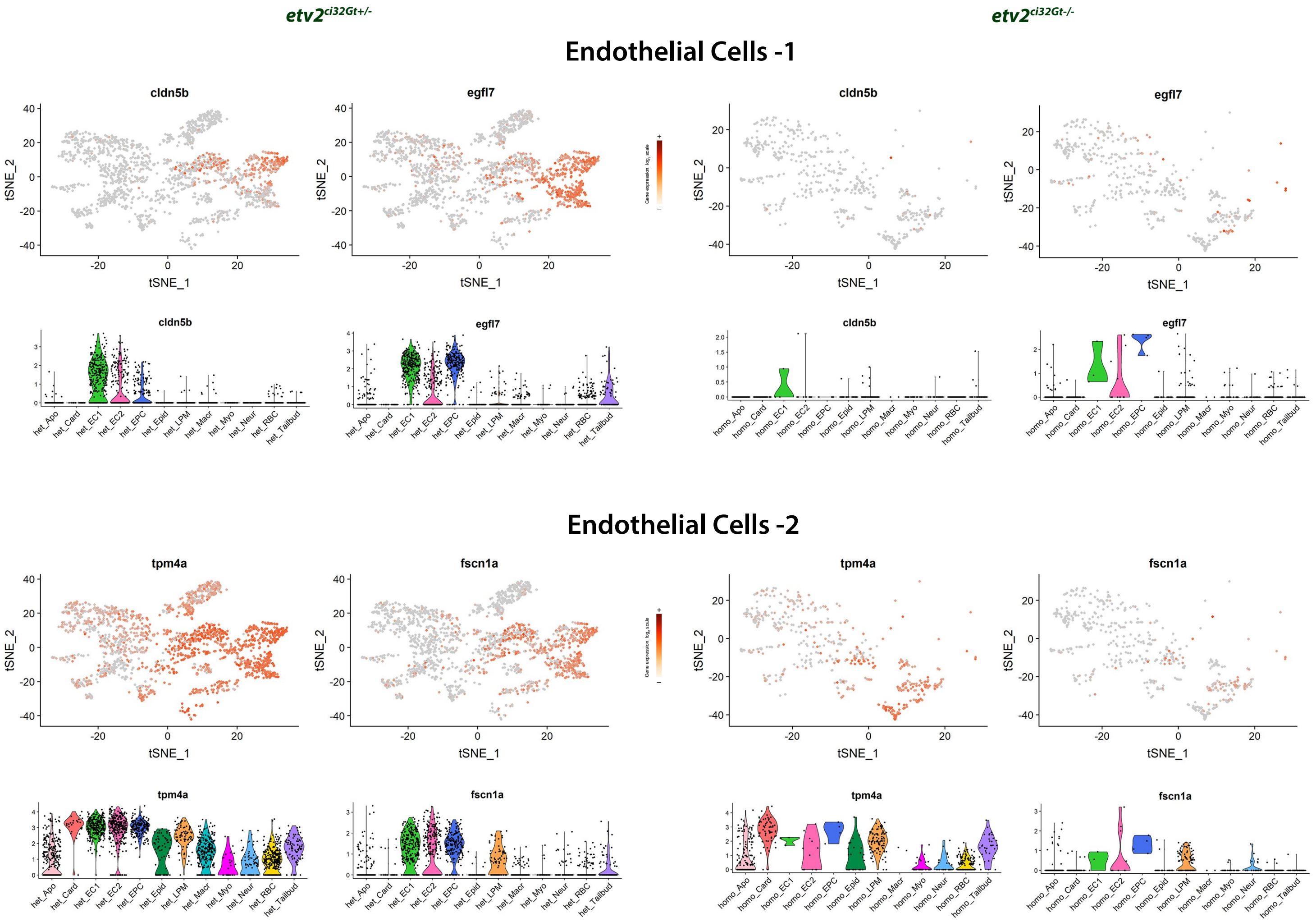

Supplementary Figure 3. t-SNE and Violin plots showing expression of selected marker genes for EC-1 and EC-2 cells in different cell populations in *etv2*$^{ci32Gt+/-}$*; UAS:GFP* and *etv2*$^{ci32Gt-/-}$*; UAS:GFP* embryos.

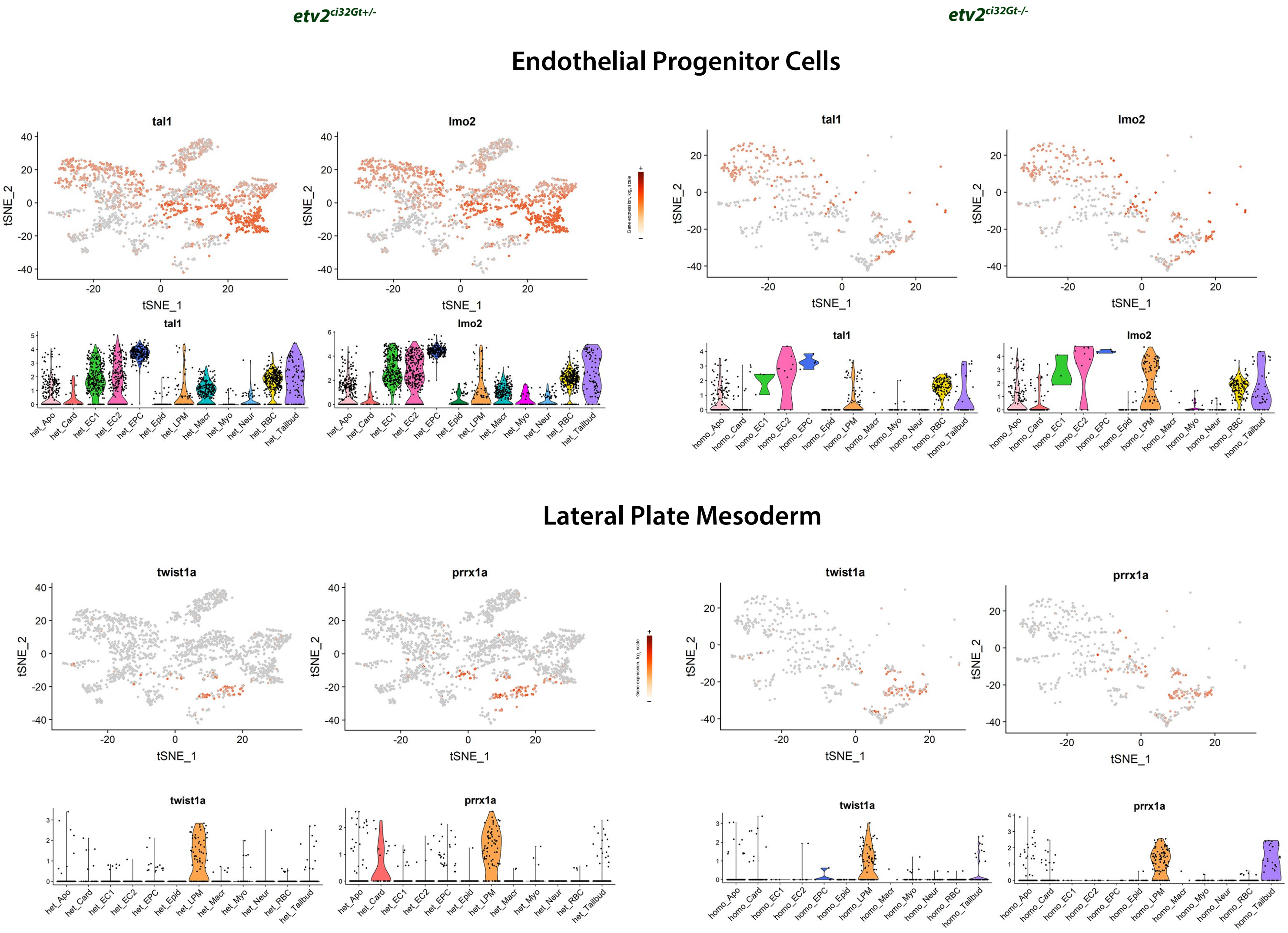

Supplementary Figure 4. t-SNE and Violin plots showing expression of selected marker genes for endothelial progenitor cells and lateral plate mesoderm in different cell populations in *etv2$^{ci32Gt+/-}$; UAS:GFP* and *etv2$^{ci32Gt-/-}$; UAS:GFP* embryos.

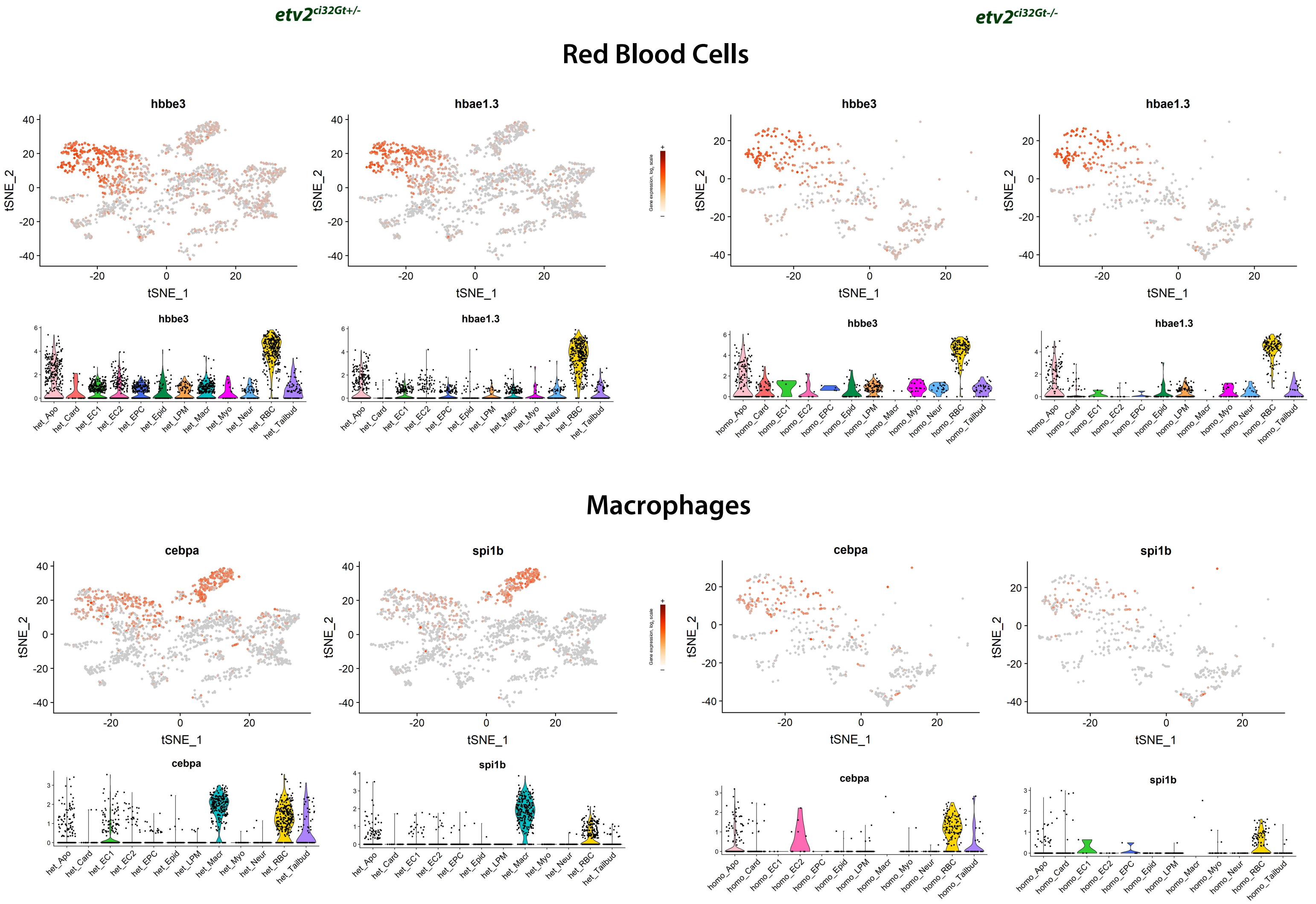

Supplementary Figure 5. t-SNE and Violin plots showing expression of selected marker genes for red blood cells and macrophages in different cell populations in *etv2$^{ci32Gt+/-}$; UAS:GFP* and *etv2$^{ci32Gt-/-}$; UAS:GFP* embryos.

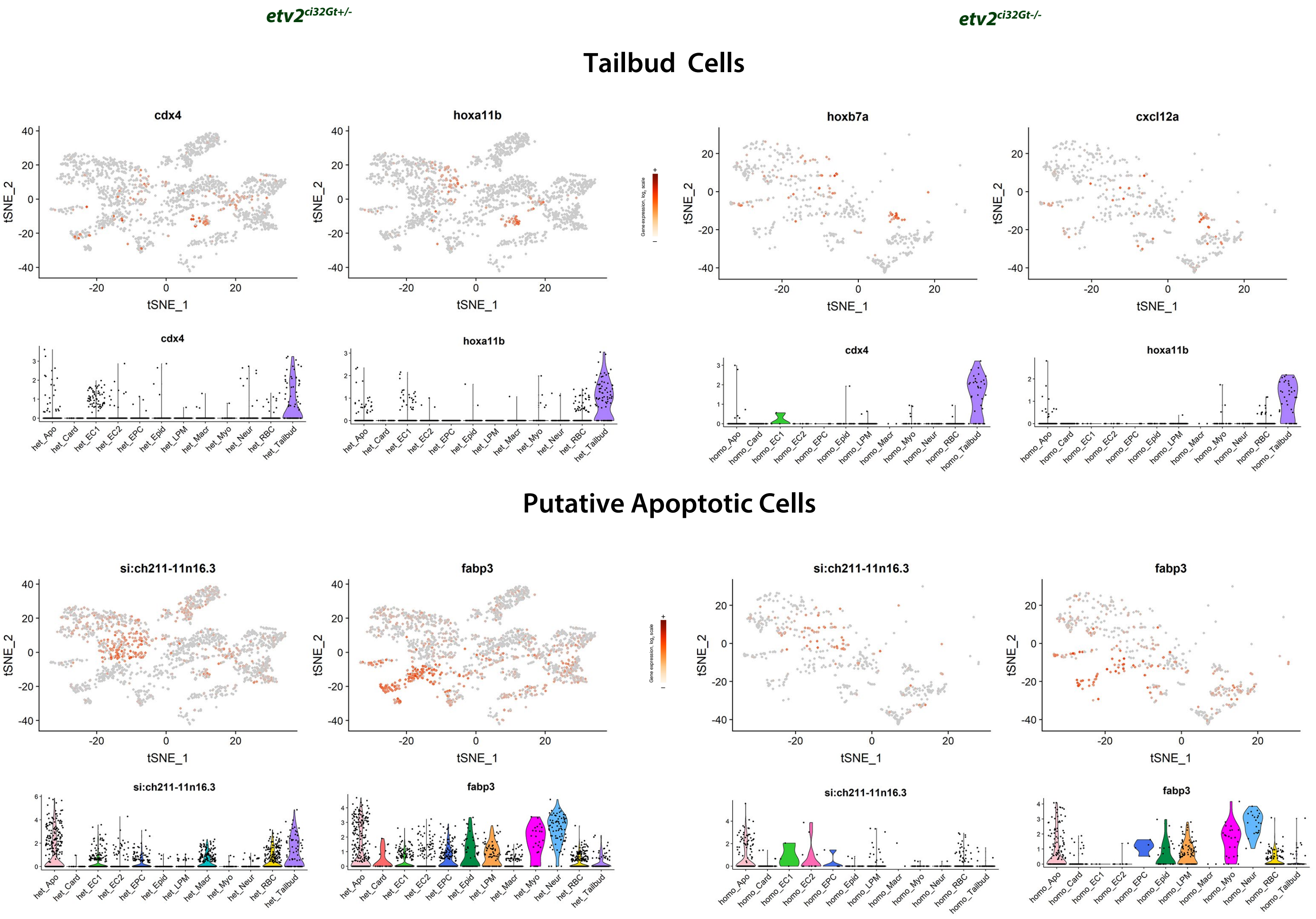

Supplementary Figure 6. t-SNE and Violin plots of expression of selected marker genes for tailbud derived progenitor cells and putative apoptotic cells in different cell populations in *etv2*$^{ci32Gt+/-}$*; UAS:GFP* and *etv2*$^{ci32Gt-/-}$*; UAS:GFP* embryos.

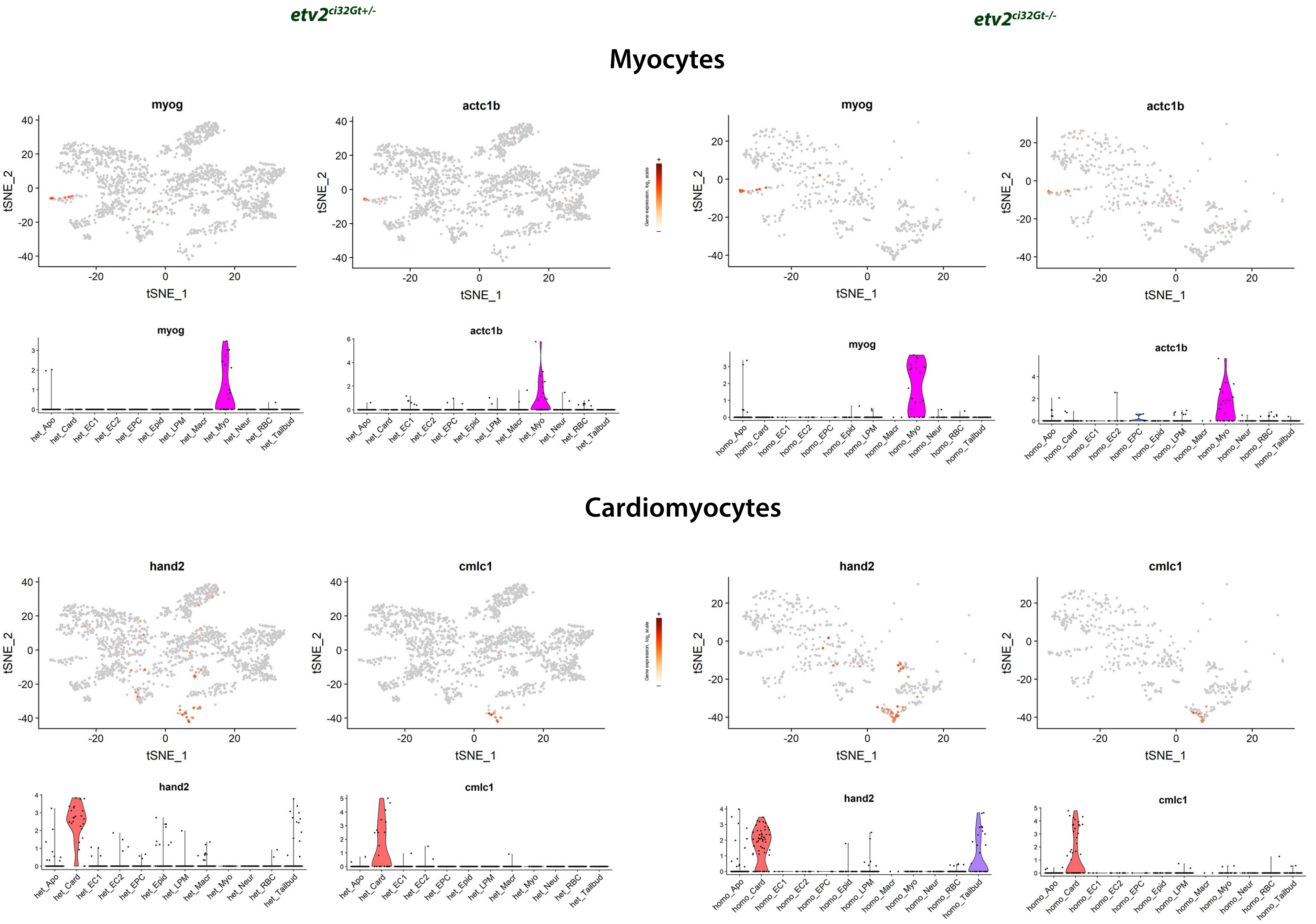

Supplementary Figure 7. t-SNE and Violin plots of expression of selected marker genes for myocytes and cardiomyocytes in different cell populations in *etv2$^{ci32Gt+/-}$; UAS:GFP* and *etv2$^{ci32Gt-/-}$; UAS:GFP* embryos.

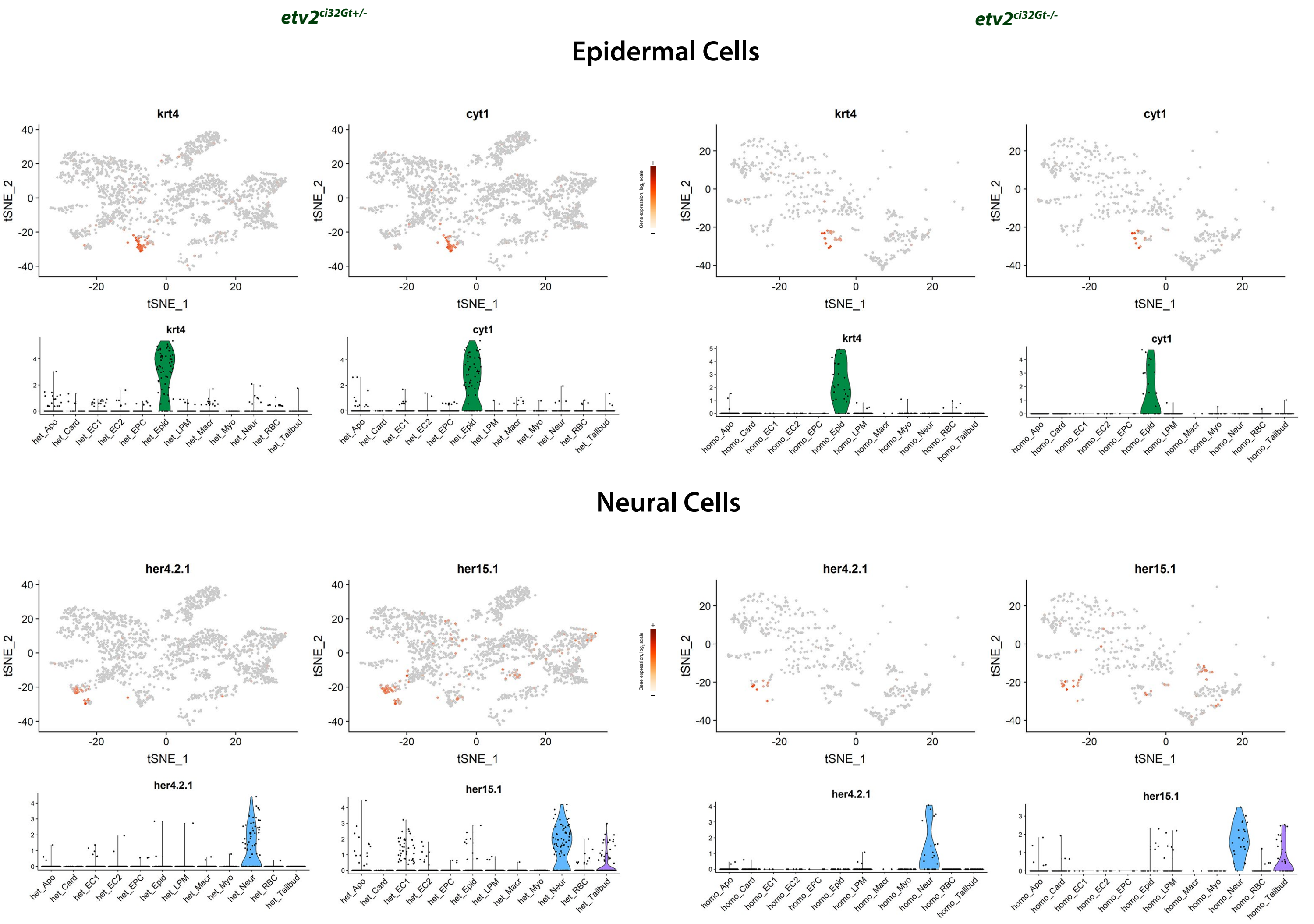

Supplementary Figure 8. t-SNE and Violin plots of expression of selected marker genes for epidermal and neural cells in different cell populations in *etv2*$^{ci32Gt+/-}$*; UAS:GFP* and *etv2*$^{ci32Gt-/-}$*; UAS:GFP* embryos.

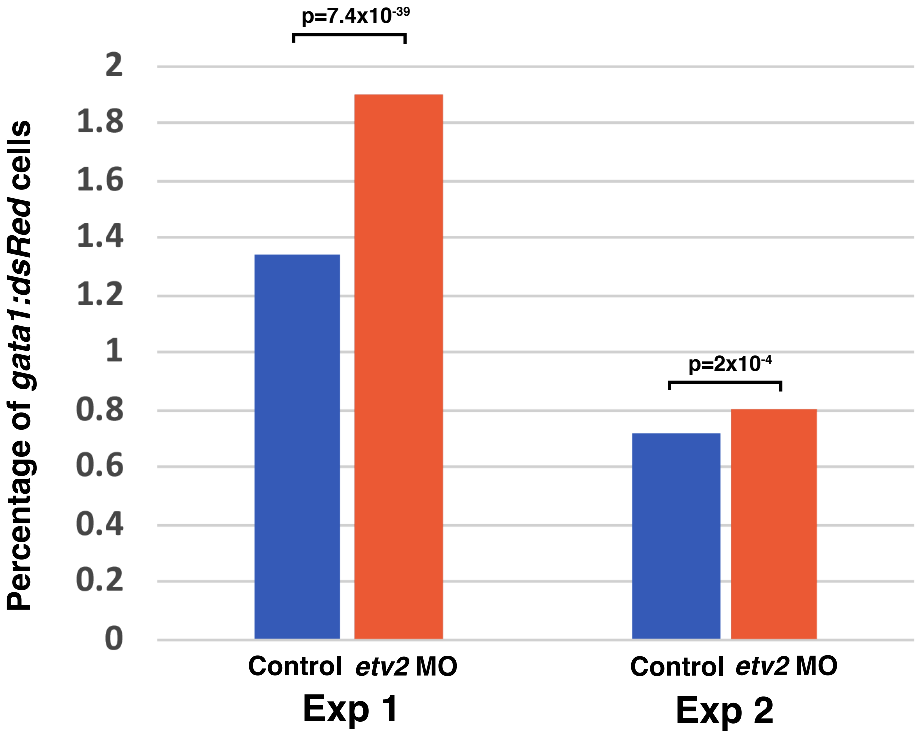

Supplementary Figure 9. Analysis of *gata1:dsRed*-positive cells in *etv2* MO-injected embryos and uninjected controls at 23 hpf. Cells from 50-70 embryos in each group were disaggregated and analyzed by FACS in two independent experiments (Exp 1 and Exp 2). Percentage of *gata1:dsRed*-positive red blood cells out of the total number of cells is shown. chi-square test was used for p calculations, n= 210,618 and 150,661 total cells in control and *etv2* MO embryos, respectively, in Exp1 and 341,808 and 265,078 cells, respectively, in Exp 2.

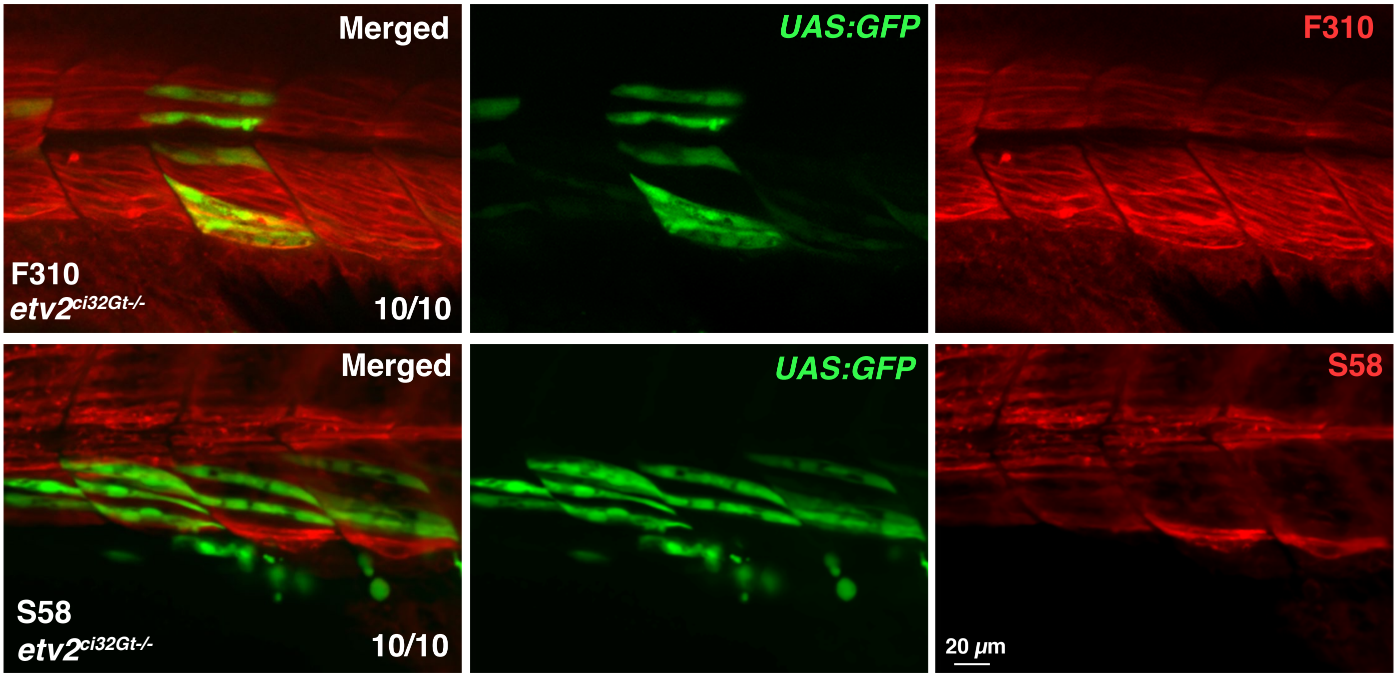

Supplementary Figure 10. Immunostaining for fast muscle actin (F310) or slow muscle actin (S58) in ***etv2*** $^{ci32Gt-/-}$***; UAS:GFP* embryos at 24 hpf.** Embryos were imaged using confocal microscopy. Maximum-intensity projection of selected confocal slices is shown. Note the overlap between GFP and F310 immunostaining and no overlap with S58 staining. Lateral view, anterior is to the left. The number of embryos displaying the representative phenotype out of the total number of embryos is shown in the lower right corner. Two replicate experiments were performed.

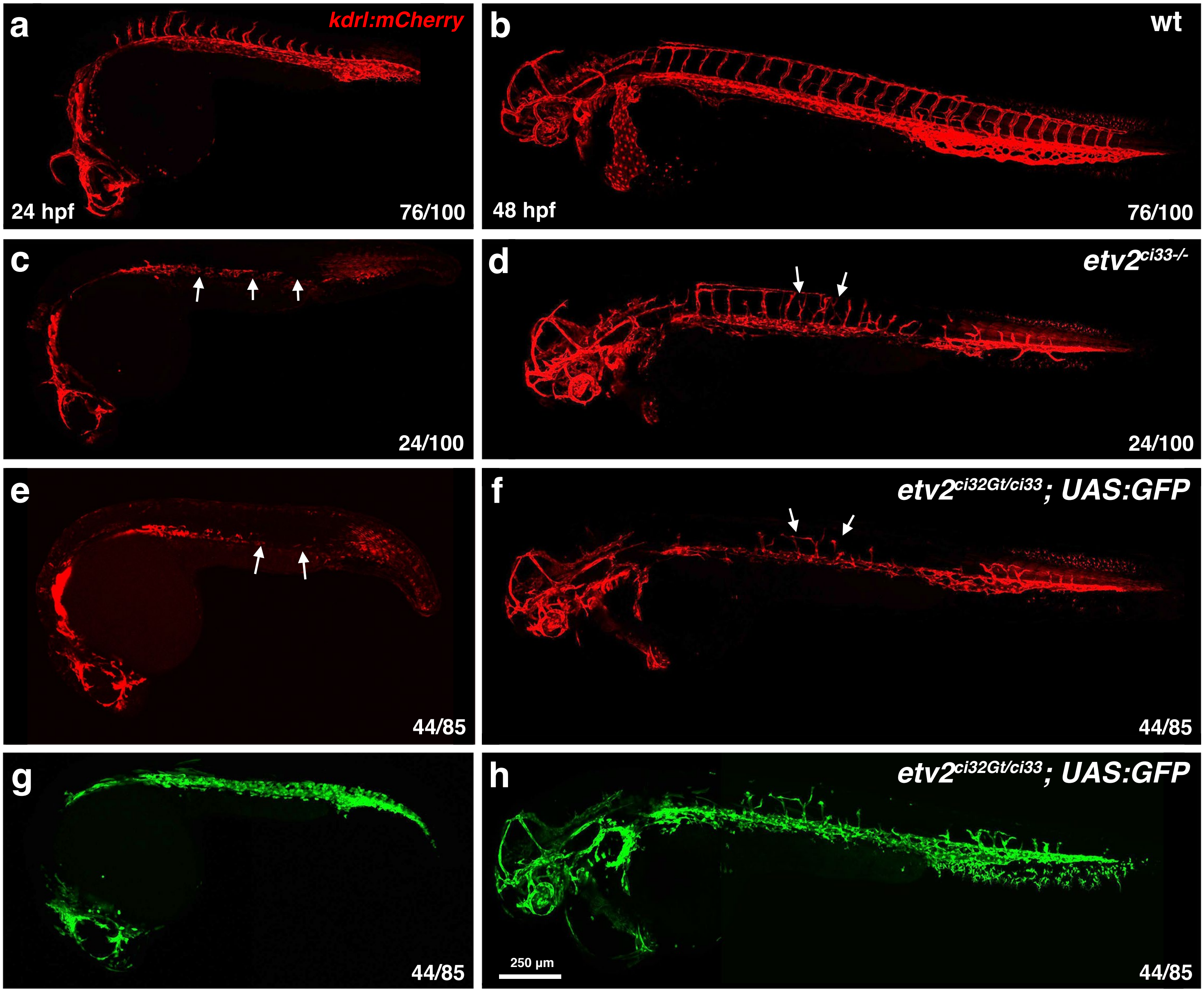

Supplementary Figure 11. Vascular defects in *etv2^{ci33}* mutants. (a-d) *etv2$^{ci33-/-}$; kdrl:mCherry* embryos show reduced mCherry expression, no intersegmental vessels and gaps (arrows, **c**) in axial vessel formation at 24 hpf, and mispatterned (arrows, **d**) as well as truncated and missing ISVs at 48 hpf. **(e-h)** Double heterozygous *etv2$^{ci32Gt/ci33}$; UAS:GFP; kdrl:mCherry* embryos show gaps in axial vasculature at 24 hpf (arrows, **e**), and absent and truncated ISVs at 48 hpf (arrows, **f**). The same embryos in red and green channels are shown. Note that vascular defects in *etv2$^{ci32Gt/ci33}$* embryos are more severe compared to the homozygous *etv2^{ci33}* mutants. In all panels, the numbers in the lower left corner display the number of embryos showing the expression pattern out of the total number of fluorescent embryos analyzed in two replicate experiments. Note that approximately 25% of embryos show the mutant phenotype in **a-d**, while 50% of GFP+ embryos are expected to carry the *ci33* allele and show the phenotype in **e-h.**

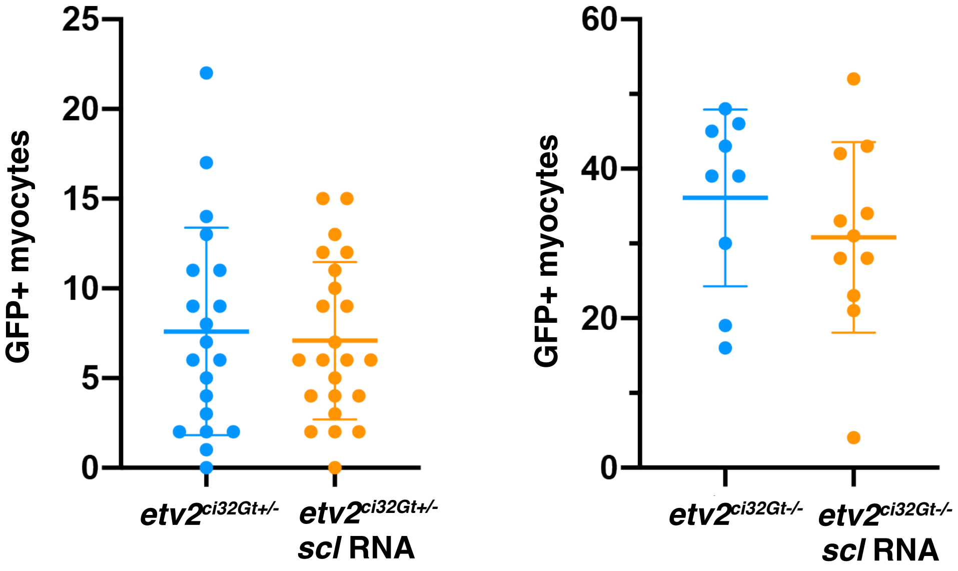

Supplementary Figure 12. Injection of *scl* mRNA does not affect the number of GFP+ skeletal muscle cells in *etv2*^{ci32Gt} heterozygous or homozygous embryos at 24 hpf. No significant difference between *scl* RNA-injected and uninjected sibling embryos was observed (p=0.74 for heterozygous and 0.35 for homozygous embryos, respectively, Student's two-tailed t-test). 20 control and 23 *scl* RNA-injected *etv2*$^{ci32Gt+/-}$ embryos, and 9 control and 11 *scl* RNA-injected *etv2*$^{ci32Gt-/-}$ embryos were analyzed in two replicate experiments. Median ± SD values are shown.

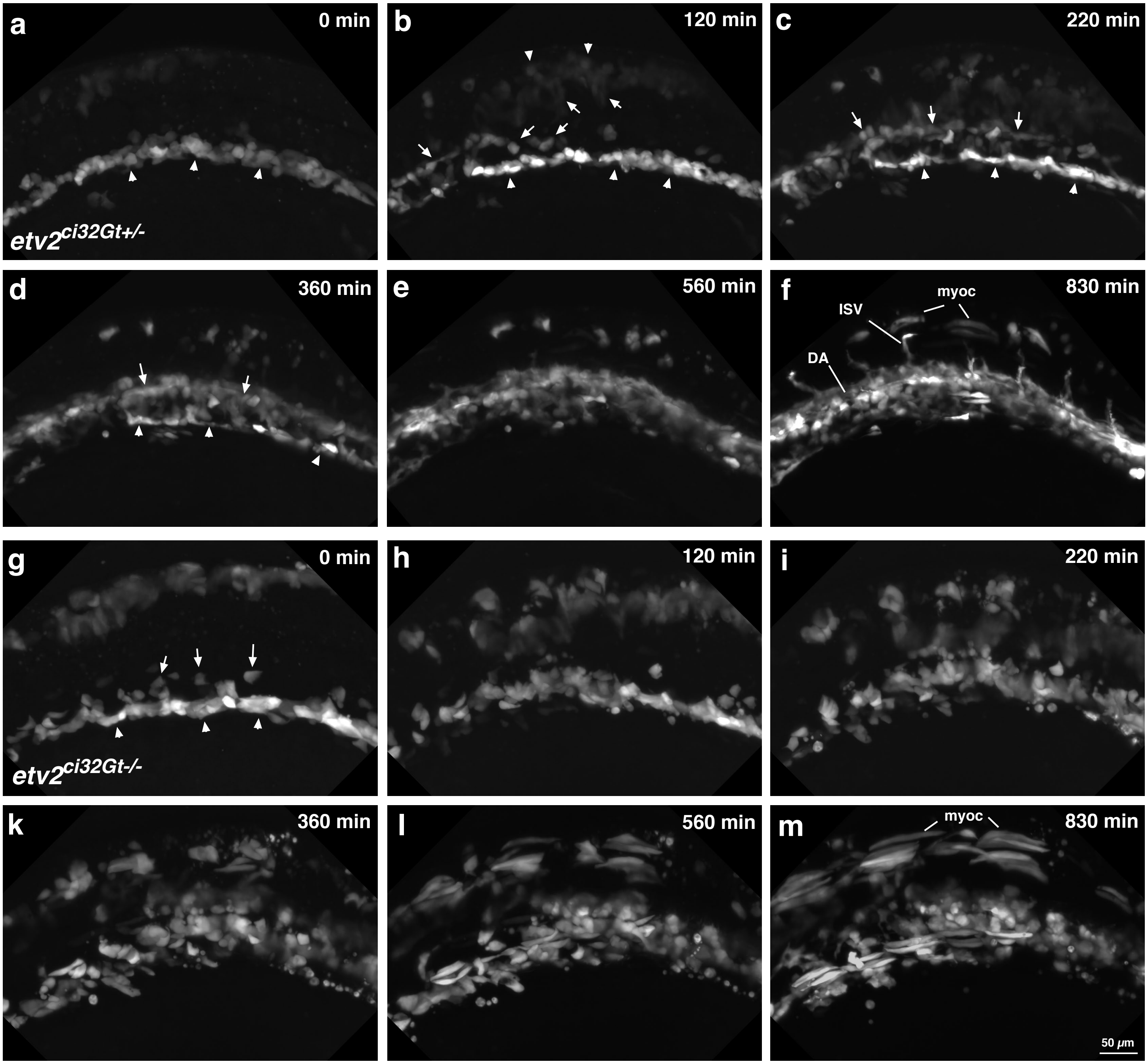

Supplementary Figure 13. Time-lapse imaging of cell migration in *etv2^{ci32Gt}; UAS:GFP* heterozygous and homozygous embryos starting at the 9-10-somite stage. Dorsolateral view is shown, anterior is to the left.
(a-f) In *etv2$^{ci32Gt+/-}$; UAS:GFP* embryos, bilaterally located vascular and hematopoietic progenitors within the lateral plate mesoderm (LPM, arrowheads) migrate towards the midline and coalesce into the axial vasculature (arrows). Note that some cells stay in the lateral position and elongate into muscle cells (myoc). DA, progenitors of the dorsal aorta, ISV, intersegmental vessels. Time frames are selected from the Movie 2. **(g-l)** In *etv2$^{ci32Gt-/-}$; UAS:GFP* embryos cells initiate migration (arrows) but fail to coalesce into the axial vasculature. Instead, many cells differentiate into myocytes (myoc). Time frames are selected from the Supplementary Movie 5. Representative embryos are shown out of the total of 6 heterozygous and 2 homozygous *etv2^{ci32Gt}*; UAS:GFP embryos that were imaged in two replicate experiments.

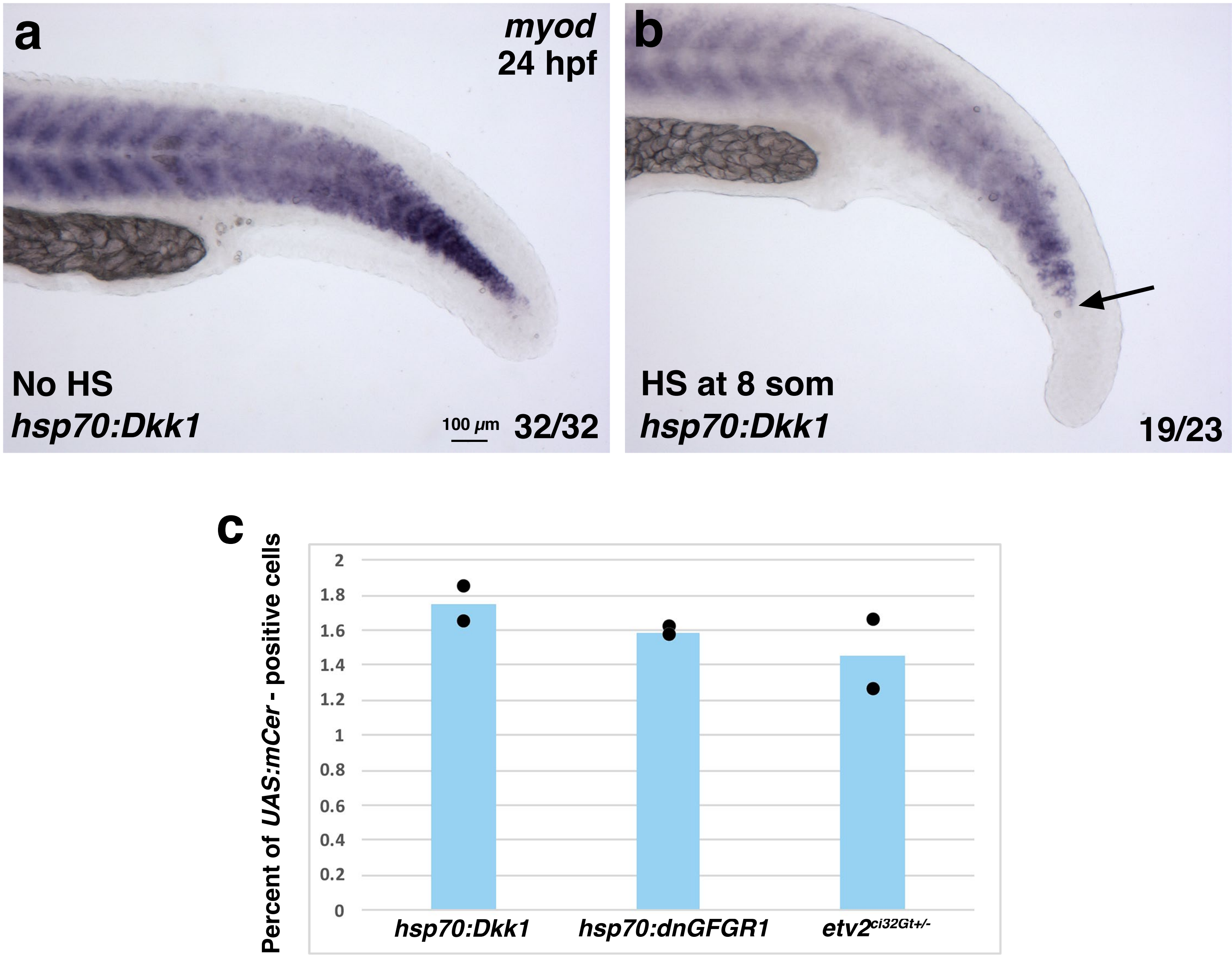

Supplementary Figure 14. Analysis for *myod* expression in *hsp70:Dkk1* embryos and percentage of fluorescent cells in *hsp70:Dkk1* and *hsp70:dnFGFR1* embryos. (a,b) In situ hybridization analysis for *myod* expression in *hsp70:Dkk1* embryos at 24 hpf. Heat-shock was performed at the 8-somite stage. Note the absence of *myod* expression in the tailbud region (arrow, b) in Dkk1-overexpressing embryos.

(c) Percentage of fluorescent cells which express the *etv2* reporter was estimated in *etv2*$^{ci32Gt+/-}$*; UAS:mCerulean (mCer)* embryos alone or crossed with *hsp70:Dkk1-GFP* and *hsp70:dnFGFR1-GFP* lines. *UAS:mCer* line was used to distinguish expression from GFP present in *hsp70* lines. Heat-shock was performed at the 8-somite stage. In a single experiment, cells from 10-25 embryos in each group were disaggregated at 24 hpf and *mCer*-positive cells were sorted by FACS to estimate their numbers. Values from two technical replicates (cell counts from 15,000-30,000 cells) are shown.

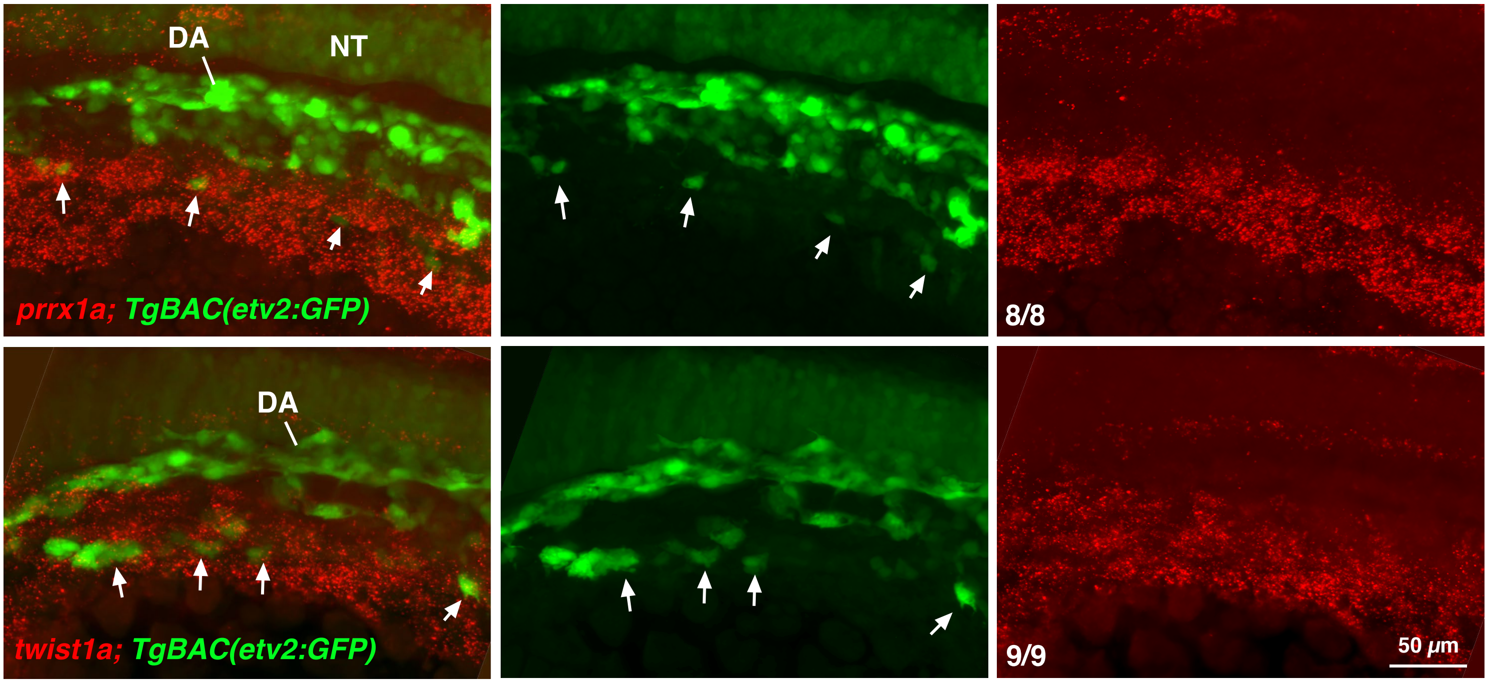

Supplementary Figure 15. Expression of *prrx1a* and *twist1a* in wild-type *TgBAC(etv2:GFP)* embryos at the 18-somite stage. Note an overlap between GFP and *prrx1a* or *twist1a* expression in the most laterally positioned GFP+ cells (arrows). DA, dorsal aorta, NT, neural tube. Dorsolateral view of the anterior trunk portion of an embryo, anterior is to the left. The numbers in the lower left corner display the number of embryos showing the expression pattern out of the total number of fluorescent embryos analyzed in two replicate experiments.

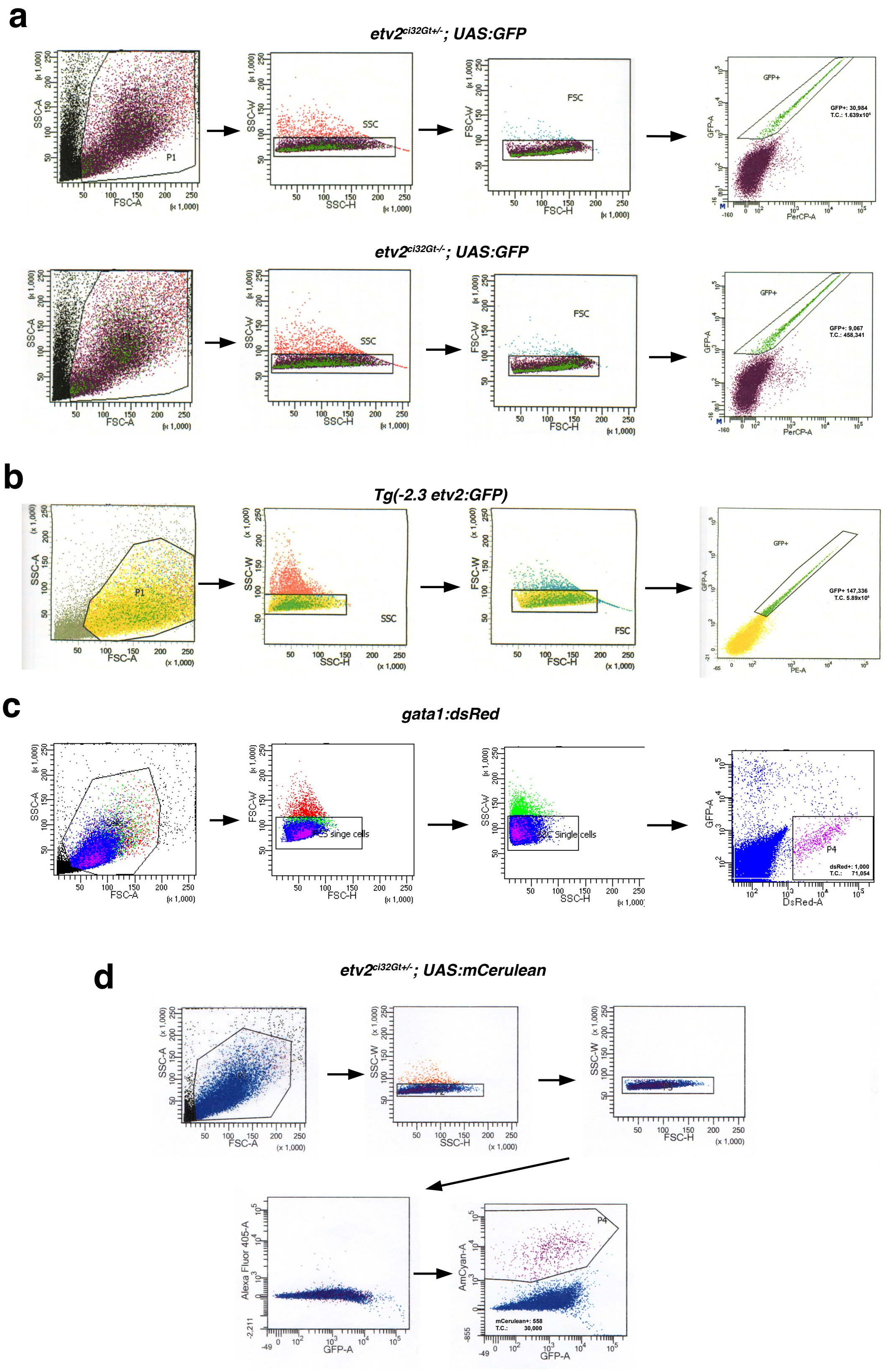

Supplementary Figure 16. Diagrams of FACS gating strategies. (a) Strategy used to sort *etv2*$^{ci32Gt+/-}$*; UAS:GFP* and *etv2*$^{ci32Gt-/-}$*; UAS:GFP* cells used for the Chromium single-cell analysis, shown in Fig. 1. **(b)** Strategy used to sort *Tg(-2.3 etv2:GFP)* cells used for the Fluidigm single-cell analysis, shown in Fig. 8. **(c)** Strategy used to sort dsRed-positive cells from *gata1:dsRed* embryos, shown in Supplementary Fig. 9. **(d)** Strategy used to sort mCerulean-positive cells from *etv2*$^{ci32Gt+/-}$*; UAS:mCerulean*-positive embryos, shown in Supplementary Fig. 14c. Am-Cyan channel was used to separate mCerulean cells from GFP fluorescence present in *hsp70:Dkk1-GFP* and *hsp70:dnFGFR1-GFP* lines. Gating strategies for representative samples are shown. Fluorescent cell numbers out of the total count (T.C.) are shown for representative samples.

Gene	average log FC	p value adjusted
	**1 - Cardiomyocytes**	
smtnl1	2.86	8.8E-224
**cmlc1**	2.72	1.4E-150
**myl7**	2.54	1.9E-276
tnni1b	2.48	0.0E+00
tnnt2a	2.28	4.6E-117
**hand2**	2.14	1.5E-180
rbm24a	1.92	2.2E-43
ttn.2	1.85	1.9E-80
podxl	1.84	8.5E-50
tnnc1a	1.80	7.4E-193
	**2 - Endothelial Cells-1**	
**cldn5b**	1.40	8.4E-180
**egfl7**	1.39	6.0E-159
sox7	1.31	2.5E-152
ecscr	1.28	6.9E-145
dusp5	1.28	3.1E-113
si:ch211-156j16.1	1.24	3.1E-115
msna	1.19	5.1E-104
crip2	1.17	5.5E-104
hey2	1.16	8.9E-130
**cdh5**	1.15	2.1E-204
	**3 - Endothelial Cells-2**	
**crip2**	1.08	2.3E-28
si:ch211-156j16.1	1.04	2.5E-27
**tpm4a**	0.90	2.3E-51
**fscn1a**	0.78	2.2E-20
erg	0.73	1.2E-12
dusp5	0.73	2.9E-31
fli1a	0.73	3.0E-14
ramp2	0.72	8.2E-07
rhoca	0.72	4.0E-12
krt18	0.72	2.9E-31
	**4 - Endothelial Progenitor Cells**	
**tal1**	1.97	3.0E-112
**lmo2**	1.97	1.9E-106
**etv2**	1.76	6.9E-141
tmem88a	1.69	7.3E-116
si:dkey-52l18.4	1.67	1.2E-131
egfl7	1.55	4.6E-121
sox7	1.42	1.5E-115
serpinh1b	1.18	9.8E-83
dusp5	1.11	1.6E-74
klf6a	1.10	2.6E-90
	**5 - Epidermis**	
**krt4**	3.61	6.2E-205
**cyt1**	3.28	3.1E-183
cfl1l	3.02	4.1E-170
cyt1l	2.94	1.1E-203
tmsb1	2.76	2.0E-69
cldni	2.57	4.7E-147
icn	2.07	9.6E-09
pfn1	1.98	7.0E-22
s100a10b	1.97	9.0E-44
tagln2	1.84	1.5E-40
	**6 - Lateral Plate Mesoderm**	
**id3**	1.66	6.8E-77
jdp2b	1.34	3.8E-52
**twist1a**	1.21	6.5E-159
**prrx1a**	1.15	1.1E-173
pitx3	1.07	3.0E-173
si:ch211-286o17.1	1.05	6.7E-125
si:ch73-335l21.4	1.02	5.5E-47
cdh11	0.94	1.7E-75
foxp4	0.94	2.4E-99
mfap2	0.93	4.0E-65

Gene	average log FC	p value adjusted
	**7 - Macrophages**	
**cebpb**	2.08	3.4E-264
**spi1b**	1.79	1.6E-219
pfn1	1.53	1.5E-120
MFAP4 (1 of many)	1.50	4.8E-183
kctd12.2	1.50	3.1E-186
txn	1.44	3.9E-119
fn1a	1.42	1.7E-161
cotl1	1.40	3.8E-123
si:zfos-741a10.3	1.38	0.0E+00
**cebpa**	1.27	3.2E-122
	**8-Myocytes**	
**actc1b**	2.84	2.0E-98
**myog**	2.17	7.3E-234
tnni2b.1	2.08	1.7E-39
**myod1**	2.08	0.0E+00
tcf15	1.94	2.1E-83
rbm24a	1.63	2.1E-54
vwde	1.55	1.1E-74
efemp2b	1.44	7.5E-84
hsp90aa1.1	1.26	5.1E-122
rprmb	1.20	1.7E-79
	**9 - Neural cells**	
si:ch211-193l2.5	2.62	1.3E-289
si:ch211-193l2.3	2.26	0.0E+00
**her4.2.1**	2.09	9.5E-214
si:ch211-193l2.4	2.03	0.0E+00
**her15.1**	1.97	5.2E-138
her4.4	1.80	4.4E-272
elavl3	1.77	6.7E-222
fabp3	1.73	9.0E-56
her15.1.1	1.68	5.9E-140
her12	1.63	1.1E-70
	**10 - Apoptotic cells**	
**si:ch211-11n16.3**	1.63	8.2E-12
**fabp3**	0.96	1.9E-03
ENSDARG00000100513	0.88	1.5E-02
tubb2b	0.60	1.1E-08
ccng1	0.38	1.0E-00
pmaip1	0.35	1.9E-06
snu13b	0.27	8.8E-02
hmgb2b	0.26	2.6E-08
	**11 - Red Blood Cells**	
**hbae1.3**	3.30	2.1E-251
hbae3	2.96	3.3E-212
**hbbe3**	2.96	2.9E-211
hbbe1.1	2.78	2.7E-202
hbbe1.3	2.40	5.6E-252
hbbe1.2	1.88	4.5E-249
cpox	1.84	1.5E-236
klf17	1.83	9.3E-304
znfl2a	1.82	3.7E-228
hbbe2	1.75	9.0E-181
	**12 - Tailbud**	
cxcl12a	1.59	1.0E-36
**hoxb7a**	1.41	7.6E-123
apoc1	1.38	4.5E-29
**cdx4**	1.37	2.2E-84
hspb1	1.37	3.2E-07
cxcl12b	1.25	2.1E-28
**hoxa11b**	1.19	2.4E-139
hes6	1.15	1.4E-07
apoeb	1.09	2.1E-30
hoxc3a	1.05	6.0E-119

Supplementary Table 1. Top marker genes for each cell population. Distribution of selected markers (shown in bold) across different cell populations is shown in t-SNE and Violin plots in Fig. 1 and Supplementary Figs. 2-8. The complete list of marker genes is shown in Supplementary Data 1.

Gene ID	p	Fold Change	Gene Symbol	Description
ENSDARG00000079078	0.002	6.88	hbaa1	hemoglobin alpha adult-1 [Source:ZFIN;Acc:ZDB-GENE-980526-79]
ENSDARG00000077231	0.005	5.09	vwf	von Willebrand factor [Source:ZFIN;Acc:ZDB-GENE-070103-1]
ENSDARG00000078004	0.012	1.56	drl	draculin [Source:ZFIN;Acc:ZDB-GENE-991213-3]
ENSDARG00000029019	0.0035	1.44	epb41b	erythrocyte membrane protein band 4.1b (elliptocytosis 1, RH-linked) [Source:ZFIN;Acc:ZDB-GENE-030130-1]
ENSDARG00000079305	0.022	1.41	hbae3	hemoglobin alpha embryonic-3 [Source:ZFIN;Acc:ZDB-GENE-990706-3]
ENSDARG00000045143	0.053	1.39	hbbe2	hemoglobin beta embryonic-2 [Source:ZFIN;Acc:ZDB-GENE-040702-1]
ENSDARG00000089963	0.047	1.35	hbbe1.1	hemoglobin beta embryonic-1.1 [Source:ZFIN;Acc:ZDB-GENE-030616-7]
ENSDARG00000006818	8.14E-04	1.35	urod	uroporphyrinogen decarboxylase [Source:ZFIN;Acc:ZDB-GENE-000208-18]
ENSDARG00000089475	0.037	1.35	hbae1	hemoglobin alpha embryonic-1 [Source:ZFIN;Acc:ZDB-GENE-980526-80]
ENSDARG00000038147	0.039	1.30	hbbe3	hemoglobin beta embryonic-3 [Source:ZFIN;Acc:ZDB-GENE-980526-287]

Supplementary Table 2. Differential expression analysis of RBC-specific genes from the global RNA-seq transcriptome analysis of *etv2*^{ci32Gt} homozygous versus heterozygous embryos at 24 hpf.
