# Supplementary material for: Single-cell transcriptomic analysis identifies the conversion of zebrafish Etv2-deficient vascular progenitors into skeletal muscle
Source: Nat Commun. 2020 Jun 3;11:2796. doi: 10.1038/s41467-020-16515-y (PMC7271194; doi:10.1038/s41467-020-16515-y)
Supplement: Supplementary file 14 — Description of Additional Supplementary Files [file 41467_2020_16515_MOESM14_ESM.pdf]

Title: Supplementary Movie 1.
Description: Time-lapse imaging of cell migration in an *etv2$^{ci32Gt+/-}$; UAS:GFP* heterozygous embryo starting at the 9-10-somite stage. Lateral view is shown, anterior is to the left. Bilaterally located vascular and hematopoietic progenitors within the lateral plate mesoderm migrate towards the midline and coalesce into the axial vasculature. Selected time frames from this movie are shown in Fig. 4a-f.

Title: Supplementary Movie 2.
Description: Time-lapse imaging of cell migration in an *etv2$^{ci32Gt+/-}$; UAS:GFP* heterozygous embryo starting at the 9-10-somite stage. Dorsolateral view is shown, anterior is to the left. Bilaterally located vascular and hematopoietic progenitors within the lateral plate mesoderm migrate towards the midline and coalesce into the axial vasculature. Selected time frames from this movie are shown in Supplementary Fig. 13a-f.

Title: Supplementary Movie 3.
Description: Time-lapse imaging of cell migration in an *etv2$^{ci32Gt-/-}$; UAS:GFP* homozygous mutant embryo starting at the 9-10-somite stage. Lateral view is shown, anterior is to the left. Cells initiate migration but fail to coalesce into the axial vasculature. Instead, many cells either undergo apoptosis or differentiate into myocytes. Selected time frames from this movie are shown in Fig. 4g-l.

Title: Supplementary Movie 4.
Description: Higher magnification view of an *etv2$^{ci32Gt-/-}$; UAS:GFP* embryo showing differentiation of a GFP+ progenitor cell initially positioned in the LPM into a myocyte (arrow). The embryo is the same as shown in Supplementary Movie 3. Selected time frames from this movie are shown in Fig. 4m.

Title: Supplementary Movie 5.
Description: Time-lapse imaging of cell migration in an *etv2$^{ci32Gt-/-}$; UAS:GFP* homozygous embryo starting at the 9-10-somite stage. Dorsolateral view is shown, anterior is to the left. Cells initiate migration but fail to coalesce into the axial vasculature. Instead, many cells differentiate into myocytes. Selected time frames from this movie are shown in Supplementary Fig. 13g-l.

Title: Supplementary Data 1.
Description: Expression of marker genes in different cell populations identified by droplet (Chromium) scRNA-seq analysis of *etv2^{ci32Gt}; UAS:GFP* embryos.

Title: Supplementary Data 2.
Description: Expression of all genes in different cell populations. Average $\log_2$ values are shown.

Title: Supplementary Data 3.
Description: Differentially expressed genes in the red blood cells of *etv2^{ci32Gt}*

homozygous embryos compared to heterozygous embryos. Increased RBC-specific genes in homozygous embryos are shown in bold.

Title: Supplementary Data 4.
Description: Significantly enriched genes in the endocardial cell cluster versus the remaining EC1 cells (EC1a cluster).

Title: Supplementary Data 5.
Description: Expression of marker genes in different cell populations identified by Fluidigm scRNA-seq analysis of *etv2:GFP* embryos.

Title: Supplementary Data 6.
Description: Expression of all genes in different cell populations identified by Fluidigm cell sorting of *etv2:GFP* embryos.
