# Supplementary material for: Single-cell transcriptomic analysis identifies the conversion of zebrafish Etv2-deficient vascular progenitors into skeletal muscle
Source: Nat Commun. 2020 Jun 3;11:2796. doi: 10.1038/s41467-020-16515-y (PMC7271194; doi:10.1038/s41467-020-16515-y)
Supplement: Supplementary file 15 — Reporting Summary [file 41467_2020_16515_MOESM15_ESM.pdf]

nature research

Corresponding author(s): Saulius Sumanas

Last updated by author(s): Apr 1, 2020

Reporting Summary

Nature Research wishes to improve the reproducibility of the work that we publish. This form provides structure for consistency and transparency in reporting. For further information on Nature Research policies, see Authors & Referees and the Editorial Policy Checklist .

Statistics

For all statistical analyses, confirm that the following items are present in the figure legend, table legend, main text, or Methods section.

n/a	Confirmed	
☐	☒	The exact sample size (*n*) for each experimental group/condition, given as a discrete number and unit of measurement
☐	☒	A statement on whether measurements were taken from distinct samples or whether the same sample was measured repeatedly
☐	☒	The statistical test(s) used AND whether they are one- or two-sided<br>*Only common tests should be described solely by name; describe more complex techniques in the Methods section.*
☐	☒	A description of all covariates tested
☐	☒	A description of any assumptions or corrections, such as tests of normality and adjustment for multiple comparisons
☐	☒	A full description of the statistical parameters including central tendency (e.g. means) or other basic estimates (e.g. regression coefficient) AND variation (e.g. standard deviation) or associated estimates of uncertainty (e.g. confidence intervals)
☐	☒	For null hypothesis testing, the test statistic (e.g. *F*, *t*, *r*) with confidence intervals, effect sizes, degrees of freedom and *P* value noted<br>*Give P values as exact values whenever suitable.*
☒	☐	For Bayesian analysis, information on the choice of priors and Markov chain Monte Carlo settings
☒	☐	For hierarchical and complex designs, identification of the appropriate level for tests and full reporting of outcomes
☒	☐	Estimates of effect sizes (e.g. Cohen's *d*, Pearson's *r*), indicating how they were calculated

Our web collection on statistics for biologists contains articles on many of the points above.

Software and code

Policy information about availability of computer code

Data collection	Axiovision 4.9 (Zeiss) was used for embryo imaging on a compound microscope.<br>Nikon Elements 4.6 (Nikon) was used for image acquisition during confocal microscopy
Data analysis	Monocle 2.8.0 was used for single-cell RNA-seq analysis obtained with the Chromium system<br>Cell Ranger 2.0.0 was used for processing and de-multiplexing raw sequencing data obtained by the Chromium system<br>R version 3.5.2 and Seurat version 2.3.4 was used for analysis of scRNA-seq results obtained with the Chromium system<br>AltAnalyze 2.1.2 was used for single-cell RNA-seq analysis obtained with the Fluidigm system<br>Adobe Photoshop CS6 was used for image processing and to assemble figures.<br>StepOne Software v2.3 (Applied Biosystems) was used for qPCR analysis<br>Prism 8 (GraphPad) was used for statistical analysis and graph generation (except for scRNA-seq data)

For manuscripts utilizing custom algorithms or software that are central to the research but not yet described in published literature, software must be made available to editors/reviewers. We strongly encourage code deposition in a community repository (e.g. GitHub). See the Nature Research guidelines for submitting code & software for further information.

Data

Policy information about availability of data

All manuscripts must include a data availability statement. This statement should provide the following information, where applicable:
- Accession codes, unique identifiers, or web links for publicly available datasets
- A list of figures that have associated raw data
- A description of any restrictions on data availability

All data that support the findings of this study are available from the corresponding author upon reasonable request.
scRNA-seq data generated in the study using the Chromium (10x Genomics) platform have been deposited to NCBI GEO database under the accession number GSE143750: https://www.ncbi.nlm.nih.gov/geo/query/acc.cgi?acc=GSE143750

scRNA-seq data generated in the study using the Fluidigm platform have been deposited to NCBI GEO database under the accession number GSE142484: https://www.ncbi.nlm.nih.gov/geo/query/acc.cgi?acc=GSE142484

Field-specific reporting

Please select the one below that is the best fit for your research. If you are not sure, read the appropriate sections before making your selection.

[x] Life sciences [ ] Behavioural & social sciences [ ] Ecological, evolutionary & environmental sciences

For a reference copy of the document with all sections, see nature.com/documents/nr-reporting-summary-flat.pdf

Life sciences study design

All studies must disclose on these points even when the disclosure is negative.

Sample size	Based on our previous studies and accepted standards in the field, we typically analyzed at least 15-20 embryos in at least two replicate experiments for each assay, which is sufficient to observe a statistical difference between treatments based on t-Student's test. Practical considerations, such as how many embryos can be imaged on a confocal microscope during a limited time, limited the number of embryos that could be analyzed in some experiments.
Data exclusions	Data were not excluded from any successful experiments.
Replication	Each experiment was successfully replicated at least twice. The number of replicates for each experiment is listed in figure legends.
Randomization	Samples were randomly distributed into different groups where possible (for example DMSO and SU5402 treatments). In other cases, etv2 ci32Gt heterozygous and homozygous embryos were separated based on their phenotypes and random representative embryos were chosen for analysis.
Blinding	Blinded analysis for most experiments was not possible because treatment conditions were often easily recognizable based on the embryo phenotypes (for example, heat-shocked embryos can be recognized based on GFP induction). However, the same scoring criteria were applied for analyzing different groups of embryos.

Reporting for specific materials, systems and methods

We require information from authors about some types of materials, experimental systems and methods used in many studies. Here, indicate whether each material, system or method listed is relevant to your study. If you are not sure if a list item applies to your research, read the appropriate section before selecting a response.

Materials & experimental systems

n/a	Involved in the study
[ ]	[x] Antibodies
[x]	[ ] Eukaryotic cell lines
[x]	[ ] Palaeontology
[ ]	[x] Animals and other organisms
[x]	[ ] Human research participants
[x]	[ ] Clinical data

Methods

n/a	Involved in the study
[x]	[ ] ChIP-seq
[ ]	[x] Flow cytometry
[x]	[ ] MRI-based neuroimaging

Antibodies

Antibodies used	Antibodies against fast and slow skeletal muscle, cat No. F310 and S58, respectively, Developmental Studies Hybridoma Bank, no lot # was provided. Anti-mouse IgG, CF594 antibody (Sigma-Aldrich, SAB4600098), lot# 2096656 was used as a secondary antibody.
Validation	Antibodies produced expected staining pattern of fast and slow zebrafish skeletal muscle. Such staining pattern has been previously reported using these antibodies in multiple previous studies, such as Kok et al 2007, Dev Biol 307, 214-26; Elworthy et al 2008; Development 135, 2115-26.

Animals and other organisms

Policy information about studies involving animals; ARRIVE guidelines recommended for reporting animal research

Laboratory animals	Zebrafish (Danio rerio) embryos at stages from 8-somite to 24 hpf were used for analysis; specific stages for each experiment are noted in the figures; sex could not been determined yet at the analyzed stages (it is specified later in development). The following strains were used in the study: Tg(5xUAS:EGFP), Tg(UAS-E1B:NTR-mCherry), Tg(etv2:mCherry)zf373, Tg(-2.3 etv2:GFP) zf372, TgBAC(etv2:GFP)ci1, etv2y11, Tg(fli1a:GFP)y1, Tg(gata1a:dsRed), Tg(actc1b:GFP), Tg(hsp70l:dkk1b-GFP), Tg

	(hsp70l:dnfgfr1a-EGFP), etv2 ci32Gt, etv2 ci33, kdrl:mCherry. Specific fish strains used are noted within each figure.
Wild animals	The study did not involve wild animals.
Field-collected samples	The study did not involve samples collected from the field.
Ethics oversight	The study was approved by the Cincinnati Children's Hospital Medical Center IACUC committee, animal protocol IACUC2016-0039

Note that full information on the approval of the study protocol must also be provided in the manuscript.

Flow Cytometry

Plots

Confirm that:

- [x] The axis labels state the marker and fluorochrome used (e.g. CD4-FITC).
- [x] The axis scales are clearly visible. Include numbers along axes only for bottom left plot of group (a 'group' is an analysis of identical markers).
- [x] All plots are contour plots with outliers or pseudocolor plots.
- [x] A numerical value for number of cells or percentage (with statistics) is provided.

Methodology

Sample preparation	To sort cells from zebrafish etv2^ci32Gt; UAS:GFP embryos for Chromium scRNA-seq, Approximately 100-150 of etv2ci32Gt+/-; UAS:GFP and 75-100 etv2ci32Gt-/-; UAS:GFP embryos were collected at the 20-somite stage and dissociated into a single-cell suspension using the previously reported protocol.63 Briefly, the embryos were manually dechorionated, and transferred into the deyolking buffer (55 mM NaCl, 1.8 mM KCl, 1.25 mM NaHCO3). Embryos were pipetted up and down on ice using p1000 pipettor until yolk was dissolved. Embryos were centrifugated at 300G for 1 min, and supernatant was removed, while the pellet was resuspended in 0.5x Danieau solution (1x Danieau: 58 mM NaCl, 0.7 mM KCl, 0.4 mM MgSO4, 0.6 mM Ca(NO3)2, 5 mM HEPES, pH 7.6). Centrifugation and removal of supernatant was repeated again, followed by another centifugation at 300G. The pellet was resuspended in FACSmax solution cell dissociation solution , which was then passed through a cell strainer. Cells were centrifugated at 300G and suspended in a buffer of 1X PBS containing 1 mM EDTA and 2% fetal bovine serum (FBS). To sort cells from Tg(-2.3 etv2:GFP) line for Fluidigm scRNA-seq, Approximately 300 Tg(-2.3 etv2:GFP) embryos were collected at each stage, dissociated into a single-cell suspension as described above and suspended in a buffer of 1X PBS containing 1 mM EDTA and 2% fetal bovine serum (FBS). To count gata1:dsRed-positive cells in wild-type and etv2 MO-injected embryos, cells from 50-70 embryos in each group were disaggregated as described above at approximately 23 hpf. To count the percentage of fluorescent cells which express etv2 reporter in estimated in etv2ci32Gt+/-; UAS:mCerulean (mCer) embryos alone or crossed with hsp70:Dkk1-GFP and hsp70:dnFGFR1-GFP lines, Cells from 10-25 embryos in each group were disaggregated at 24 hpf as described above.
Instrument	BD FACS Aria II (BD Biosciences) was used for cell sorting. BD FACS Canto II (BD Biosciences) was used to count gata1:dsRed positive cells.
Software	BD FACSDiva 8.0.1 software
Cell population abundance	Does not apply for cell counting experiments (cells are not sorted and only counted; not possible to measure post-sorting abundance). For cell sorting experiments of Tg(etv2:GFP) and etv2^ci32Gt; UAS:GFP cells, post-sorting abundance measurements were not performed in order to minimize the time before cells were processed for scRNA-seq by either Chromium or Fluidigm approaches. Instead, scRNA-seq results were validated by other approaches such as confirming expression patterns of marker genes of different cell populations as described in the manuscript.
Gating strategy	Gating strategy is shown in supplementary Figure 16. First a morphology gate (FSC versus SSC) is drawn. Then this morphology gate is reported to a 2nd window (FCS versus pulse width) to define the singulets and exclude the doublets. The singulet gate is reported to a 3rd window (FSC versus live/dead stain) to exclude the dead cells. The intersection of the morphology, singulet and live gates are then reported to all the downstream windows displaying fluorescence X and Y parameters.<br>For GFP+ cells, GFP-negative samples were used to set up GFP+ gates.<br>For gata1:dsRed cells, dsRed-negative samples were used to set up dsRed+ gates.<br>For mCerulean+ cells, AmCyan channel was used for detection, 405 nm excitation, emitation 525 ± 50 nm. 488 nm excitation and 530±30 nm emission was used for GFP detection. GFP negative and mCerulean negative samples were used to set up gates and distinguish mCerulean from GFP fluorescence.

- [x] Tick this box to confirm that a figure exemplifying the gating strategy is provided in the Supplementary Information.
